# Inhibition of Polyinosinic-Polycytidylic Acid-Induced Acute Pulmonary Inflammation and NF-κB Activation in Mice by a Banana Plant Extract

**DOI:** 10.7150/ijms.88748

**Published:** 2024-01-01

**Authors:** Chien-Huang Liao, Tung-Yuan Lai, Yu-Ying Lin, Yi-Chun Liao, Gi-Ming Lai, Tien-Hua Chu, Szu-Yao Wu, Wei-Lun Tsai, Jacqueline Whang-Peng, Frank Liu, Tzeon-Jye Chiou, Chih-Jung Yao

**Affiliations:** 1 Cancer Center, Wan Fang Hospital, Taipei Medical University, Taipei 11696, Taiwan.; 2 Department of Chinese Medicine, Hualien Tzu Chi Hospital, Buddhist Tzu Chi Medical Foundation, Hualien 97002, Taiwan.; 3 Traditional Chinese Medicine Cancer Center, Hualien Tzu Chi Hospital, Hualien 97002, Taiwan.; 4 Institute of Biomedical Sciences, Academia Sinica, Taipei, 11529, Taiwan.; 5 Division of Hematology and Medical Oncology, Department of Internal Medicine, Wan Fang Hospital, Taipei Medical University, Taipei 11696, Taiwan.; 6 Chimera Bioscience Inc., No. 18 Siyuan St., Zhongzheng Dist., Taipei 10087, Taiwan.; 7 Department of Research and Development, Natural Well Technical Company, Guishan, Taoyuan 33377, Taiwan.; 8 Department of Medical Education and Research, Wan Fang Hospital, Taipei Medical University, Taipei 11696, Taiwan.; 9 Department of Internal Medicine, School of Medicine, College of Medicine, Taipei Medical University, Taipei 11031, Taiwan.

**Keywords:** banana plant extract, acute pulmonary inflammation, IL-6, NF-κB

## Abstract

NF-κB activation is pivotal for the excess inflammation causing the critical condition and mortality of respiratory viral infection patients. This study was aimed to evaluate the effect of a banana plant extract (BPE) on suppressing NF-κB activity and acute lung inflammatory responses in mice induced by a synthetic double-stranded RNA viral mimetic, polyinosinic-polycytidylic acid (poly (I:C)). The inflammatory responses were analyzed by immunohistochemistry and HE stains and ELISA. The NF-κB activities were detected by immunohistochemistry *in vivo* and immunofluorescence and Western blot *in vitro*. Results showed that BPE significantly decreased influx of immune cells (neutrophils, lymphocytes, and total WBC), markedly suppressed the elevation of pro-inflammatory cytokines and chemokines (IL-6, RANTES, IFN-γ, MCP-1, keratinocyte-derived chemokine, and IL-17), and restored the diminished anti-inflammatory IL-10 in the bronchoalveolar lavage fluid (BALF) of poly (I:C)-stimulated mice. Accordingly, HE staining revealed that BPE treatment alleviated poly (I:C)-induced inflammatory cell infiltration and histopathologic changes in mice lungs. Moreover, immunohistochemical analysis showed that BPE reduced the pulmonary IL-6, CD11b (macrophage marker), and nuclear NF-κB p65 staining intensities, whilst restored that of IL-10 in poly (I:C)-stimulated mice. *In vitro*, BPE antagonized poly(I:C)-induced elevation of* IL-6*, nitric oxide, reactive oxygen species, NF-κB p65 signaling, and transient activation of p38 MAPK in human lung epithelial-like A549 cells. Taken together, BPE ameliorated viral mimic poly(I:C)-induced acute pulmonary inflammation in mice, evidenced by reduced inflammatory cell infiltration and regulation of both pro- and anti-inflammatory cytokines. The mechanism of action might closely associate with NF-κB signaling inhibition.

## Introduction

Viral infections can cause acute pneumonia, resulting in serious morbidity or mortality in patients with pregnancy, obesity, pre-existing chronic diseases or at extremes of age 1. Viral pneumonia is triggered by exaggerated inflammatory response associated with infiltration of leukocytes into the lung tissue, and massive production of cytokines, the so-called cytokine storm 2-4. The exaggerated inflammatory response can diffusely damage lung cells, leading to lung fibrosis and even multi-organ dysfunction 4. Current antiviral drugs are not effective when treated late after the symptoms onset 3. At present, no specific treatments are available for curing viral pneumonia 3. Infections with pneumotropic viruses, such as certain H1N1 and H5N1 influenza virus strains, severe acute respiratory syndrome coronavirus (SARS-CoV), and Middle East respiratory syndrome coronavirus (MERS-CoV) could cause lung injuries and even mortality in patients 5,6. Recently, the severe acute respiratory syndrome coronavirus 2 (SARS-CoV-2; causative agent of COVID-19) can induce critical pneumonia that may be followed by organ failure, and even death 7, which has imposed serious threats on public health burden worldwide 8. Further research for therapeutic strategies to improve the treatment outcome of these life-threatening viral pneumonias are still required and ongoing. Among the strategies proposed, the potential of anti-inflammatory therapy in the treatment of SARS-CoV-2 infection becomes a topic of great interest 9. Drugs with anti-inflammatory activity were repurposed for the treatment of COVID-19 patients although their contemporary clinical efficacy has not been proven to correlate with the current pandemic 10.

Natural products and plant extracts could be considered as a potential source to develop novel antiviral or anti-inflammatory therapeutics for treating or preventing viral pneumonia. A plethora of natural products and plant extracts were reported to exhibit potent activities against various strains of influenza virus like SARS-CoV-2 or druggable targets like angiotensin-converting enzyme 2 (ACE2) receptor 11, 12. Their potential roles in SARS-CoV-2 treatment have gained public attention recently 11, 12. In addition, natural products also contain a variety of active compounds possessing anti-inflammatory activities in repressing the crucial driven cytokine and signaling pathway such as IL-6 and NF (nuclear factor)-κB, respectively 13. Their therapeutic potential against COVID-19 inflammation also has been noticed and studied 14. *Musa paradisiaca* L., prevalently known as banana, is an herbaceous plant of the *Musaceae* family and the genus *Musa*. It is one of the most important crops of the world and is the staple food for people living in tropical region. Banana plant is rich in polyphenols, flavonoids, vitamins, potassium, magnesium, and serotonin 15-17. Compounds contained in *Musa* species had been reported to exert various biological activities, including anti-inflammation, anti-oxidation, anti-diabetes and cardiovascular protection etc. 17. The anti-inflammatory activities of *Musa* species products had been shown to reduce combined allergic rhinitis and asthma syndrome in mice 17 and attenuate cardiac hypertrophy in rats 16. Likewise, a banana plant extract (BPE) evaluated in this study was reported to promote wound healing in diabetic rats, accompanied by reduced inflammation 15. Yet, their effects on viral pneumonia inflammation remain unknown. It is rational and warranted to investigate the effect of *Musa* specie product on the relief of virus-induced acute lung inflammation and NF-κB activation, a key molecular event for eliciting inflammatory responses.

Structurally similar to double stranded RNA, polyinosinic-polycytidylic acid (poly (I:C)) can activate the Toll like receptor 3 (TLR3) pathway to mimic viral infection and induce the subsequent NF-κB activation and cytokines production, leading to pulmonary inflammation and dysfunction 18,19. This study explores the therapeutic effects of BPE on acute pulmonary inflammation induced by intranasal instillation of RNA viral mimetic poly (I:C) in mice. The impact of BPE treatment on poly (I:C)-induced infiltration of immune cells (neutrophils, lymphocytes, and total WBC), disruption of cytokines and chemokines production (IL-6, regulated on activation, normal T cell expressed and secreted (RANTES), interferon-γ (IFNγ), monocyte chemoattractant protein-1 (MCP-1), vascular endothelial growth factor (VEGF), keratinocyte-derived chemokine (KC), IL-17, and IL-10), and activation of inflammation-associated signaling pathways (NF-κB and MAPK p38) were examined.

## Materials and Methods

### Reagents

Stock solution of the banana plant extract (BPE) was provided by Natural Well Technical Ltd. Company (Guishan, Taiwan). It was diluted with culture medium for use *in vitro* and double distilled water for use *in vivo* experiments. BPE is a water-soluble substance extracted from the leaves, peels and stems of Musa paradisiaca (Linn) by a patented extraction technology (Invention patent NO. I584814, TW) as described in a previous study 15. The analyzed components contained in BPE are mainly flavone, polyphenol, polysaccharide, terpenes, and amino acids etc. More details of the components are listed in Table S1. Polyinosinic-polycytidylic acid (poly (I:C) was purchased from InvivoGen (Cat # tirl-pic, San Diego, CA) and was dissolved in phosphate-buffered saline (PBS) (10 mM phosphate, 150 mM NaCl, pH 7.4) at a concentration of 10 mg/mL and aliquots were stored at -20°C. Pyrrolidine dithiocarbamate (PDTC; an NF-κB inhibitor) was obtained from Sigma-Aldrich (Cat #P8765, St. Louis, MO, USA).

### Cell Culture

The type II pulmonary epithelial cell line A549 (BCRC 60074) was purchased from the BCRC (Bioresource Collection and Research Center, Hsin Chu, Taiwan). The human fetal lung fibroblast WI-38 and murine macrophage RAW 264.7 cells were obtained from the American Type Culture Collection (Manassas, VA, USA). A549 cells were maintained in Roswell Park Memorial Institute (RPMI)-1640 medium (Gibco, Carlsbad, CA, USA), WI-38 cells were cultured in Eagle's Minimum Essential Medium (EMEM) (Gibco), and RAW 264.7 cells were cultured in Dulbecco's Modified Eagle's Medium (DMEM) (Gibco). These culture mediums were supplemented with 10% fetal bovine serum (Gibco), 1 × penicillin streptomycin-glutamine (PSG, Gibco). Cells were cultured at 37°C in a water jacketed 5% CO2 incubator. According the effective *in vitro* concentration (1/6000) of BPE used in a previous study 15, 1/6000, 1/2000, and 1/600 dilutions were chosen as the concentrations of BPE to treat cells in this study.

### Western Blotting

After cells were seeded in 6-cm dishes at a density of 5 × 10^5^ cells/dish for 24 h, they were then pretreated with BPE for 1 h and then stimulated with poly (I:C) for the time intervals described in the legend of Figure 7. Cell extracts were prepared by resuspending cells in radioimmunoprecipitation assay (RIPA) lysis buffer containing protease inhibitor (Roche, CA, USA). After centrifugation, the supernatants were dissolved in the sample buffer for sodium dodecyl sulfate-polyacrylamide gel electrophoresis (SDS-PAGE). Samples containing equal amounts of protein (50 μg) were separated by SDS-PAGE, and then transferred onto a PVDF membrane. The membrane was blocked with 5% skim milk and probed with primary antibodies at 4°C for overnight. After incubation with horseradish peroxidase-conjugated secondary antibody (Jackson Immunoresearch, PA, USA), the membrane was then observed using Immobilon Western Chemiluminescent HRP Substrate (Millipore, MA, USA). Antibody against NF-κB p65 (sc-8088), phospho-NF-κB p65 (sc-166748), and phospho-IκBα (sc-8404) were purchased from Santa Cruz (Santa Cruz Biotechnology, Inc, CA, USA). Anti-phospho-p38 (ab-178867) and anti-GAPDH (ab-8245) were purchased from Abcam (Abcam, MA, USA). The results of Western blots were quantified by Image J software (ImageJ Version 1.52, National Institutes of Health, Bethesda, MD, USA) downloaded from https://imagej.nih.gov/ij/download.html (accessed on 25 September 2019).

### RNA extraction and Quantitative RT-PCR

After cells were seeded in 6-well plate at a density of 2 × 10^5^ cells/well for 24 h, Raw 264.7 cells were pretreated with different concentrations of BPE or PBS (Control, CTL) for 1 h, and A549 and WI-38 cells were pretreated with different concentrations of BPE, 10 μM of PDTC or PBS (Control, CTL) for 1 h. Afterward, cells were then stimulated with poly (I:C) (10 μg/mL) for 6 h and then their total RNAs were extracted using RNeasy Mini Kit and treated with an RNase-free DNase I set (Qiagen, Hilden, Germany) according to the manufacturer's protocol. Total RNA (1 μg) was reverse-transcribed using oligo (dT) 15 primers and a reverse transcription system (Promega, Madison, USA). Reactions were carried out using Fast SYBR® Green PCR Master Mix (Applied Biosystems, Warrington, UK) on the StepOne Plus Real-Time PCR System (Applied Biosystems, Foster City, CA, USA) by denaturation at 95°C for 10 min, followed by 40 cycles at 95°C for 15 s and 60°C for 40 s. Melting curve analyses were performed to verify the amplification specificity. Relative quantification of gene expression was performed according to the ΔΔ-CT method using StepOne Software 2.0 (Applied Biosystems). *Glyceraldehyde-3-phosphate dehydrogenase* (*GAPDH*) or *18S* was used to normalize the RT-PCR results. The primer sequences used were as follows:

Human *IL-6* F: 5'-GGAGAG-GAGACTTCACAGAGGA-3'; Human *IL-6* R: 5'- ATTTCCACGATTTCCCAGAGA-3'; Human *GAPDH* F: 5'-GTGGACCTGACCTGCCGTCT-3'; Human *GAPDH* R: 5'-GGAGGAGTGGGTGTCGCTGT-3'.

Mouse *IL-6* F: 5'-TGGAGTCACAGAAGGAGTGGCTAAG-3'; Mouse *IL-6* R: 5'-TCTGACCACAGTGAGGAATGTCCAC-3'; Mouse *IL-10* F: 5'-GGTTGCCAAGCCTTATCGGA-3'; Mouse *IL-10* R: 5'-ACCTGCTCCACTGCCTTGCT-3'; Mouse *18S* F: 5'-GTAACCCGTTGAACCCCATT-3'; Mouse *18S* R: 5'-CCATCCAATCGGTAGTAGCG-3'.

### Immunofluorescence analysis

For the examination of nuclear translocation of NF-κB p65, A549 cells (1.0 × 10^5^ cells/ well) were cultured in a 24-well plate and pretreated with BPE (1/6000 dilution) for 1 h and then poly (I:C) (10 μg/mL) for 4 h. Cells were fixed in 4% paraformaldehyde/PBS for 15 min at room temperature, permeabilized with 0.5% Triton X-100 in PBS for 5 min, and then blocked with 5% bovine serum albumin (BSA)/PBS for 30 min. Plate was incubated at 4 °C with anti-p65 or anti-phospho-p65 antibody. After overnight incubation, cells were washed and incubated for 1 h at room temperature with 1:2000 dilution of Alexa 488 or 555-labeled donkey anti-rabbit antibody (Life Technologies, Eugene, OR, USA). Cells were then counterstained with 4′,6-diamidino-2-phenylindole (DAPI) for cell nuclei and observed by Olympus IX73 inverted fluorescence microscope (Olympus, MA, USA). The images of immunofluorescence were analyzed by SPOT Advanced Imaging software ver5.2 (SPOT imaging, MI, USA).

### Measurement of nitrite and intracellular reactive oxygen species (ROS) levels

A549 or WI-38 cells (2 × 10^4^ cells per well in a 24-well plate) were pretreated with BPE for 1 h and then stimulated with poly (I:C) (10 μg/mL) for 24 h. The supernatants of the cultured cells were collected, and the accumulated nitrite (NO2^-^, one of two primary stable and nonvolatile breakdown products of nitric oxide (NO)) was measured using Griess reagent (Promega, Madison, WI, USA). The intracellular accumulation of ROS was measured with DCFDA / H2DCFDA-Cellular ROS Assay Kit (ab113851, Abcam) according to the manufacturer's instructions. In brief, A549 or WI-38 cells were pretreated with BPE for 1 h, then stimulated with poly (I:C) (10 μg/mL) for 24 h, and then stained with 20 mM H2DCF-DA in PBS for 1 h at 37°C. DCF fluorescence intensity was measured at 485-nm excitation and 535-nm emission using a fluorescence plate reader (Molecular Devices, CA, USA).

### Mice

Thirty-five female C57BL/6 mice (8-12 weeks old) were purchased from National Laboratory Animal Center and housed in the specific pathogen-free animal facility of Chimera Bioscience (Taipei, Taiwan). All mice experiments were followed the guidelines of the Care and Use of Laboratory Animals 20 and approved by protocol number # CMR-AP109092901 of the Institutional Animal Care and Use Committee of Chimera Bioscience (Taipei, Taiwan).

### Establishment of poly (I:C)-induced lung inflammation model

The poly (I:C)-induced lung inflammation model was established as described in a previous study 18. High molecular weight polyinosine-polycytidylic acid (HMW poly (I:C), Invivogen, San Diego, CA, USA) was prepared according to vendor's instruction. Since preliminary experiment showed that BPE at dilution of 1/60 (200 μL/mouse) was effective against poly (I:C) (100 μg/mouse)-induced pulmonary inflammation. Female C57BL/6 mice (8-12 weeks old) were equally divided to five groups (control group, poly (I:C) group, and three poly (I:C) + BPE (1/600, 1/200, 1/60 dilutions) groups). In each group, three mice were used for BALF acquisition and four mice were for immunohistochemistry (IHC) study after treatment. Mice were anesthetized with 4% isoflurane for daily intranasal instillation of poly (I:C). HMW poly (I:C) (100 μg/mouse) or PBS with a total volume of 50 μL were administered intranasally (i.n.) for consecutive 3 days with a 24-hour interval between each administration. BPE (at concentrations of 1/600, 1/200, and 1/60 dilutions) were given twice a day by oral gavage (p.o.) (200 μL/mouse) at 1 h before and 6 h after each daily poly (I:C) challenge for 3 days. Control and poly (I:C) groups were administrated with same volume of PBS (i.n.) and double-distilled water (p.o.) as poly (I:C) and BPE, respectively. Mice were then sacrificed with the overdose anesthetics Zoletil and Rompun at 24 h after the last poly (I:C) administration, and the BALF and lung were collected for further analysis.

### Collection of bronchoalveolar lavage fluid (BALF)

BALF sample was collected by flushing through the trachea back and forth for 3 times with 1 mL cold PBS. The lung tissues were then harvested and frozen. BALF was centrifuged at 500 × g for 10 min at 4 °C. Cell-free supernatants were collected and stored at -80°C until the measurement of cytokines and chemokines. The BALF cell pellet was resuspended in PBS for immune cell count by an automated haematology analyser ProCyte (IDEXX ProCyte Dx, IDEXX Laboratories, Westbrook, ME, USA).

### Histopathological examination

Mice lungs were fixed in 10% neutral buffered formalin for paraffin embedding. The whole lung was then sectioned at 5 µm and stained with hematoxylin and eosin (H&E). Histological analyses were focus on inflammatory cells infiltration and semi-quantitatively graded in a blinded manner on a range of 0 to 5 as described in a previous study 21.

### Antibody array detection of cytokines levels in BALF

Murine interleukin-6 (IL-6), IL-10, IL-17, keratinocyte-derived chemokine (KC), monocyte chemoattractant protein-1 (MCP-1), regulated upon activation normal T expressed and secreted (RANTES), vascular endothelial growth factor (VEGF), and interferon-gamma (IFN-γ) in the BALF samples were quantified with Quantibody mouse cytokine array kit (QAM-CYT-1, RayBiotech, Inc., Norcross, USA). The assay was performed according to the manufacturer's protocol and data were analyzed with the software provided by the company.

### Immunohistochemistry Assay

Immunohistochemistry (IHC) was performed as reported by a previous study 22. At 24 h after the last poly (I:C) administration, the lung tissues of mice were embedded in paraformaldehyde for the IHC staining of CD11b, IL-6, IL-10, and p65. The images of IHC were captured with Olympus BX51 microscope (Olympus, MA, USA).

### Statistical analysis

The statistical analysis of data was performed using one-way ANOVA followed by Dunnett's t test. Probability value of p < 0.05 was considered statistically significant. Single asterisk (*) indicates p < 0.05; double asterisks (**) indicate p < 0.01; triple asterisks (***) indicate p < 0.001; number sign (#) indicates p < 0.01.

## Results

### BPE decreased the elevated immune cell count in BALF from poly (I:C)-treated mice

**Figure 1:**
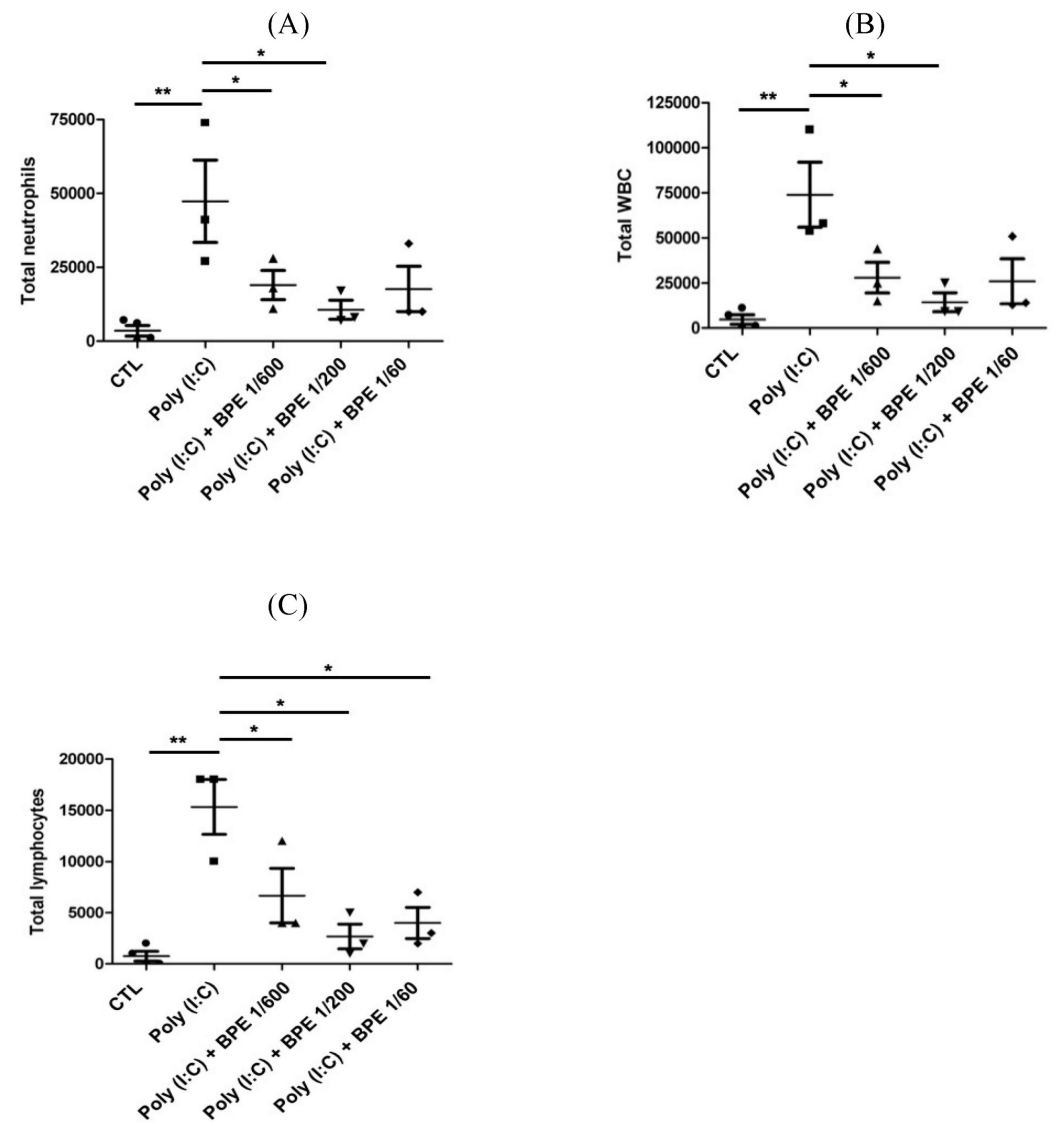
Treatment with BPE protected the influx of inflammatory cells into the airways of poly (I:C)-treated mice. C57BL/6 mice were treated with BPE (200 μL/mouse at concentration of 1/600, 1/200, 1/60 dilution) via oral gavage 1 h prior to and 6 h after an i.n. administration of poly (I:C) (100 μg in 50 μL PBS/mouse) every 24 h for three cycles. Control (CTL) group was administrated with same volume of PBS. One day after the last administration of poly (I:C), mice were euthanized and bronchoalveolar lavages (BALs) were performed. The total numbers of (A) WBC cells, (B) neutrophils, and (C) lymphocytes in the bronchoalveolar lavage fluid (BALF) were measured. The values are expressed as the means ± SD (n = 3). The value of poly (I:C) alone-treated group was compared with that of PBS-treated control (CTL) or BPE-treated groups. *p < 0.05; **p < 0.01.

To evaluate the anti-inflammatory effects of BPE *in vivo*, acute pulmonary inflammation in mice was generated by intranasal administration of poly (I:C) as described in Materials and Methods section. One day after the last poly (I:C) stimulation, severely increased immune cell number was observed in the bronchoalveolar lavage fluid (BALF) from poly (I:C) alone-treated mice, in comparison with that from PBS-treated control (CTL) (Figure 1). Treatment with BPE significantly reduced the poly (I:C)-induced elevation of total white blood cell (WBC) (Figure 1A), neutrophils (Figure 1B), and lymphocytes (Figure 1C) counts in BALF.

**Figure 2:**
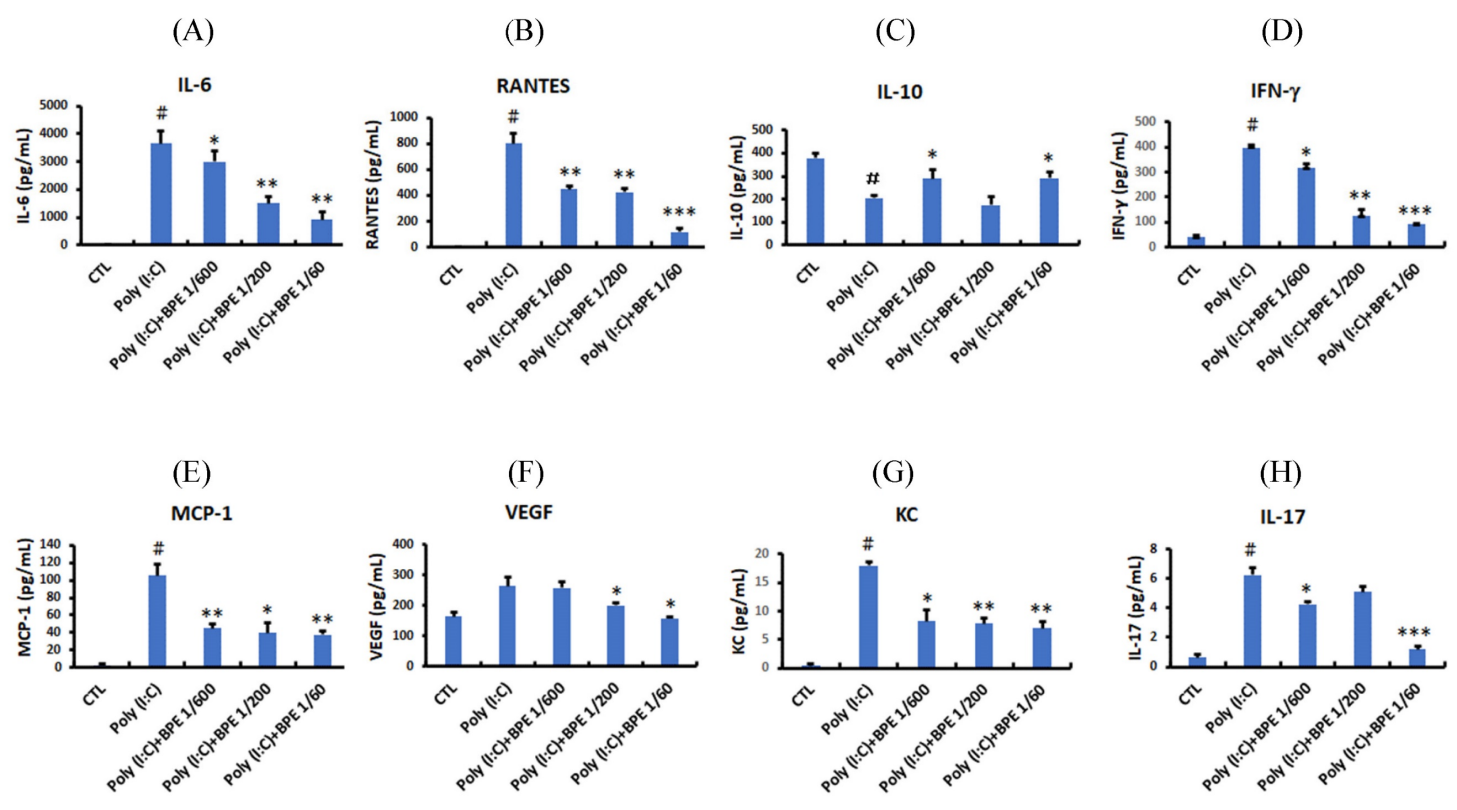
Effects of BPE on the cytokines and chemokines contented in the BALF of Poly (I:C)-stimulated mice. Cytokines and chemokines levels of (A) interleukin (IL)-6, (B) Chemokine ligand 5 (CCL5; RANTES), (C) IL-10, (D) interferon gamma (IFN-γ), (E) monocyte chemotactic protein (MCP)-1, (F) vascular endothelial growth factor (VEGF), (G) keratinocytes-derived chemokine (KC), and (H) IL-17 in the BALF of treated mice were analyzed at 24 h after the last poly (I:C) administration. Values are mean ± SD (n = 3). #p < 0.01, compared with the control (CTL) group. The values of BPE-treated groups were compared with that of poly (I:C) alone-treated group. *p < 0.05; **p < 0.01; ***p < 0.001.

Consistent with the elevated immune cell count, enzyme linked immunosorbent assay (ELISA) showed that pro-inflammatory cytokines and chemokines such as IL-6, RANTES, IFN-γ, MCP-1, KC, and IL-17 were drastically increased in the BALF from poly (I:C) alone-treated mice (Figure 2), whereas the anti-inflammatory IL-10 was diminished (Figure 2C). Treatment with BPE counteracted this phenomenon by reducing the above elevated pro-inflammatory cytokines (Figure 2), and restoring the diminished anti-inflammatory IL-10 (Figure 2C). These results show the anti-inflammatory property of BPE and imply the attenuated pulmonary inflammation in BPE-treated mice.

### BPE attenuated poly (I:C)-induced lung inflammation and histopathological changes in mice

**Figure 3:**
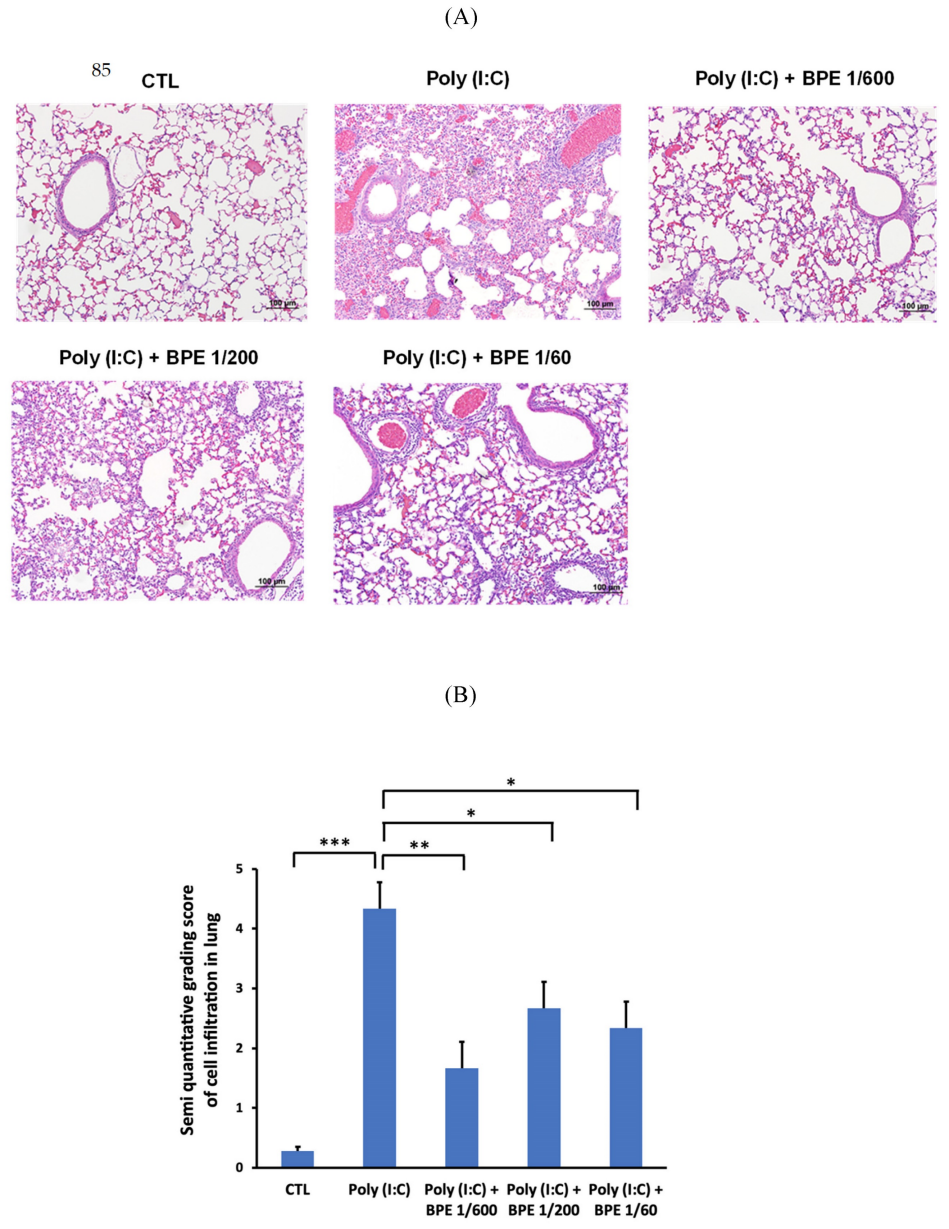
Treatment with BPE reduced poly (I:C)-caused histopathologic changes in mice. (A) Representative hematoxylin-eosin (HE) staining images showing the histopathologic changes in mice lungs of indicated groups. Mice of each group were treated as described in Materials and Methods. Lungs from each experimental group were processed for histological evaluation at 24 h after the last poly (I:C) stimulation. Scale bar = 100 μm. (B) Semi-quantitative grading score of inflammatory cells infiltration in a blinded manner on a range of 0 to 5 as described in Materials and Methods. The data are presented as the mean ± SD (n = 4). The value of poly (I:C) alone-treated group was compared with that of PBS-treated control (CTL) or BPE-treated groups. *p < 0.05; **p < 0.01; ***p < 0.001.

To confirm the effects of BPE in BALF, hematoxylin-eosin (HE) staining of the mice lung tissues was performed to examine the histopathological change. As shown in Figure 3A, HE staining showed intact bronchial and alveolar structures in the lungs of PBS-treated control mice, and no obvious inflammatory cell infiltration or interstitial hyperemia was displayed. In contrast, inflammation cells permeating into pulmonary alveoli, thickening of the alveolar wall, and pulmonary congestion were found in the lung tissues of poly(I:C) alone-treated mice (Figure 3A). In line with the results shown in BALF, BPE treatment markedly ameliorated the aforementioned poly (I:C)-induced damages, evidenced by less severe alveolar wall collapse and decreased inflammatory cell infiltration (Figure 3A). The degree of cell infiltration was semi-quantitatively scored as shown in Figure 3B. Poly (I:C) stimulation severely increased the score from 0.28 ± 0.07 of control to 4.33 ± 0.44, which could be significantly reduced to 1.66 ± 0.43, 2.66 ± 0.41, and 2.33 ± 0.40 by treatment with BPE at dilution of 1/600, 1/200, and 1/60, respectively (Figure 3B). Although the effect was not in a dose-dependent manner, BPE appeared to exhibit protective effects against the damage produced by poly (I:C) in mice lungs.

### BPE suppressed CD11b and IL-6, whilst restored IL-10 levels in poly (I:C)-stimulated mice lungs

**Figure 4:**
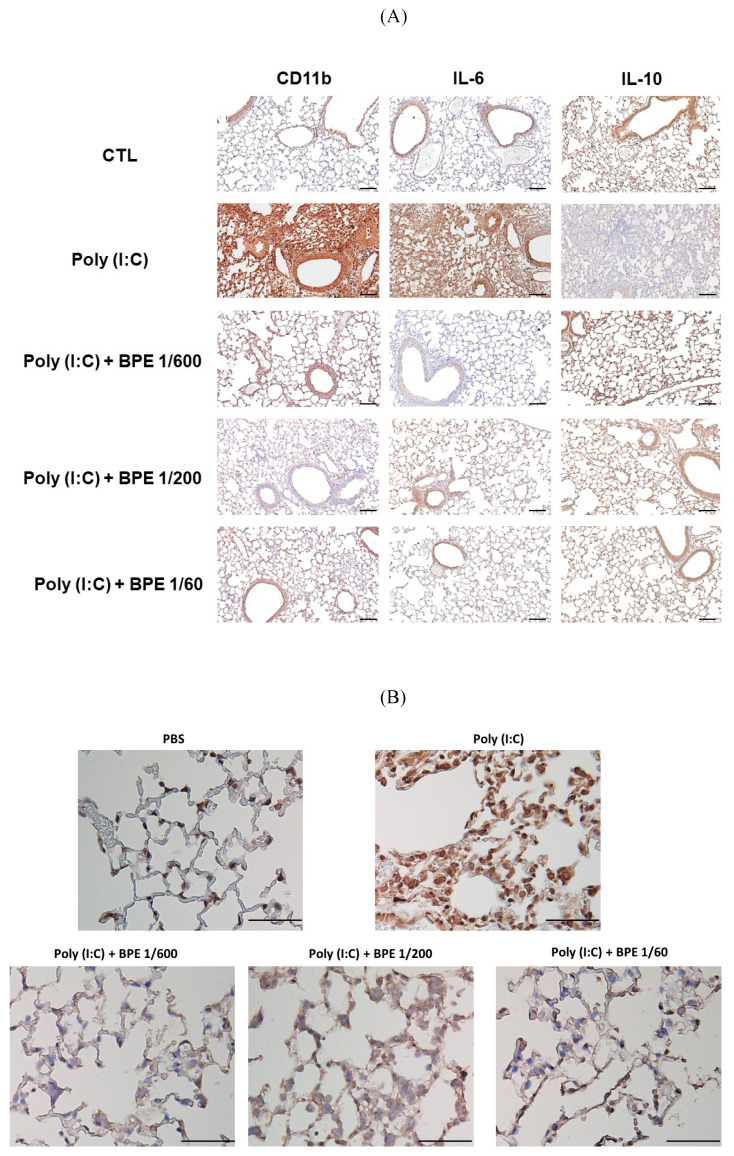
Effect of BPE on poly (I:C)-induced changes in CD11b, IL-6, IL-10, and NF-κB p65 protein levels of mice lungs. Representative immunohistochemistry staining images of (A) CD11b, IL-6, IL-10, and (B) NF-κB p65, respectively, in the lungs of mice treated as indicated. Mice of each group were treated as described in Materials and Methods. The intensity of brown positive staining represents the protein level detected by antibody used for each sample. (A) bar =100 μm, (B) bar =50 μm.

Since HE staining showed inhibitory effects of BPE on poly (I:C)-induced inflammatory cell infiltration and pathological change in mice lungs, we examined the pulmonary levels of CD11b (a marker ubiquitously expressed on monocytes and neutrophils and discretely expressed on inflammatory alveolar macrophages in mice 23), IL-6 (a crucial pro-inflammatory cytokine), and IL-10 (an important anti-inflammatory cytokine) in mice lung tissues by immunochemistry staining. In agreement with the marked inflammatory cell infiltration shown in HE staining, much higher staining intensity of CD11b, was observed in the lungs of poly (I:C) alone-stimulated mice, in comparison to that of PBS-treated control (Figure 4A). Treatment with BPE obviously reduced the intensity of CD11b staining caused by poly (I:C) stimulation (Figure 4A). In line, poly (I:C) stimulation resulted in marked increase of IL-6 and diminishment of IL-10 staining intensities, when compared with that of PBS-treated control (Figure 4A). BPE treatment reduced the elevated IL-6 whilst restored the diminished IL-10 staining intensities mentioned above (Figure 4A). These results further display the anti-inflammatory activity of BPE in this poly (I:C)-induced acute pulmonary inflammation mouse model.

### BPE suppressed poly (I:C)-induced NF-κB activation in mice lungs

In the progression of acute pulmonary inflammation, nuclear factor κB (NF-κB) activation had been demonstrated as a key event by several studies 24-26. The critical event for activating NF-κB signaling is nuclear translocation of the p65 subunit of NF-κB (NF-κB p65) 27. To investigate the mechanism underlying the BPE-exerted anti-inflammatory effects, immunohistochemistry staining was also performed to examine the nuclear translocation of NF-κB p65 protein. As expected, much higher staining intensity of nuclear NF-κB p65 was found in the lungs of poly (I:C) alone-treated mice, in comparison with that of PBS-treated control (Figure 4B). In support of its effects mentioned above, BPE treatment substantially reduced the staining intensity of nuclear NF-κB p65 protein resulted from challenge of poly (I:C) (Figure 4B). It is very likely that inhibition of NF-κB played a crucial role in the anti-inflammatory property of BPE.

### BPE suppressed poly(I:C)-induced inflammation-associated responses in cultured epithelium-like cells, fibroblast, and macrophage

Upon pulmonary viral infection, inflammation was raised by the complex interaction of lung tissues, immune cells (such as macrophages and neutrophils), and the secreted mediators (such as chemokines, cytokines and reactive oxygen species (ROS)) 28,29. To further characterize the anti-inflammatory property of BPE, we explored its effects in cultured macrophage-like, alveolar type II epithelium-like, and lung fibroblast cells primed with pro-inflammatory stimulator poly (I:C).

**Figure 5:**
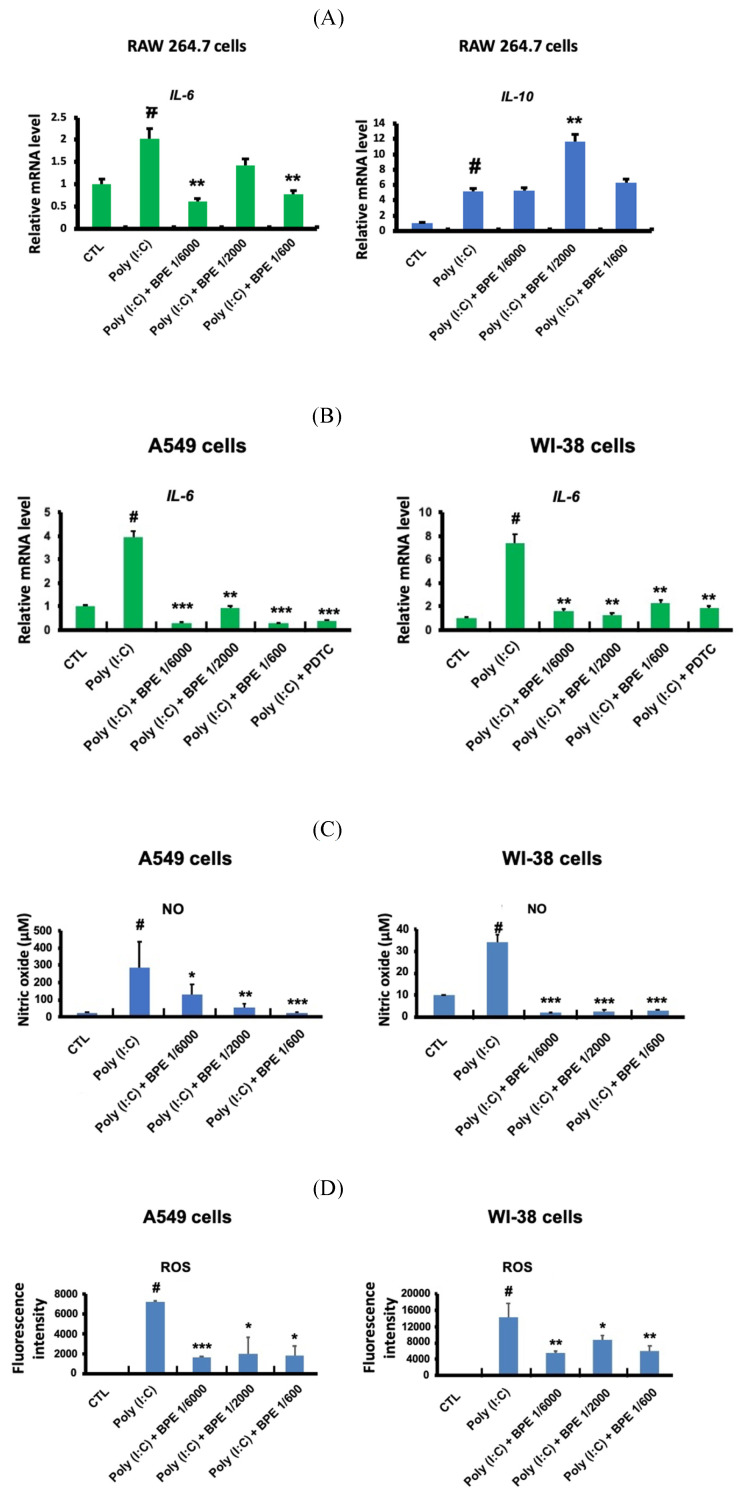
Effects of BPE on poly (I:C)-induced pro-inflammatory mediators in RAW 264.7, A549, and WI-38 cells. (A) Raw 264.7 cells were pretreated with different concentrations of BPE or PBS (Control, CTL) for 1 h and then stimulated with poly (I:C) (10 μg/mL) for 6 h. The mRNA levels of* IL-6* and* IL-10* were measured by real-time reverse-transcription polymerase chain reaction assay and normalized to 18S mRNA level. (B) A549 and WI-38 cells were pretreated with different concentrations of BPE, 10 μM of PDTC or PBS (Control, CTL) for 1 h and then stimulated with poly (I:C) (10 μg/mL) for 6 h. The mRNA level of* IL-6* was measured by real-time reverse-transcription polymerase chain reaction assay and normalized to GAPDH mRNA level. (C) A549 and WI-38 cells were pretreated with different concentrations of BPE or PBS (Control, CTL) for 1 h then incubated with poly(I:C) (10 μg/mL) for 24 h. The amount of nitric oxide (NO) released into the media was measured by Griess reagent. (D) The cellular reactive oxygen species (ROS) levels of the cells described in (C) were measured by DCFDA / H2DCFDA - Cellular ROS Assay Kit (ab113851, Abcam). The values represent mean ± SD (n=3). #p < 0.01, compared with control (CTL) group. *p < 0.05; **p < 0.01; ***p < 0.001, compared with poly (I:C) alone-treated group.

RAW 264.7 macrophage-like cells were employed to study the effects of BPE in primed macrophage. As shown in Figure 5A, poly (I:C) (10 μg/mL) significantly increased transcription of the crucial pro-inflammatory cytokine *IL-6* in RAW 264.7 cells. Pretreatment with BPE significantly suppressed the *IL-6* mRNA induction by poly (I:C), although the effect was not statistically significant at the concentration of 1/200 dilution (left panel of Figure 5A). Intriguingly, in contrast to that observed in Figure 2C and 4A, the *IL-10* mRNA level of cultured RAW 264.7 cells was elevated by challenge with poly (I:C), and treatment with BPE at concentration of 1/2000 dilution further enhanced the poly (I:C)-elevated *IL-10* transcription (right panel of Figure 5A).

A549 and WI-38 cells were employed for the experiments to study the effects of BPE in human alveolar type II epithelium-like and lung fibroblast cells, respectively. The transcription of the crucial pro-inflammatory cytokine* IL-6* in A549 and WI-38 cells was significant increased after priming with poly (I:C) (10 μg/mL) for 6 h, and pretreatment with BPE at various diluted concentrations could effectively counteract this induction of *IL-6* mRNA expression (Figure 5B). It is known that poly (I:C) induced IL-6 secretion in bronchial epithelial cells via NF-κB activation 30. An effective NF-κB inhibitor pyrrolidine dithiocarbamate (PDTC) 31 was used as a positive control to suppress the *IL-6* induction. Notably, the efficacy of BPE was similar to that of PDTC (Figure 5B). This result is in accordance with that shown in Figure 4, which implies the close association between NF-κB inhibition and the anti-inflammatory effects of BPE.

Upon priming with poly (I:C) (10 μg/mL), drastically increased productions of nitric oxide (NO) (Figure 5C) and reactive oxygen species (ROS) (Figure 5D) were detected in these A549 and WI-38 cells. Coincide with its inhibitory effect on the *IL-6* mRNA induction mentioned above, BPE also effectively suppressed poly (I:C)-induced NO and ROS productions in these A549 and WI-38 cells (Figure 5C and 5D).

### BPE suppressed poly (I:C)-induced NF-κB activation in A549 cells

**Figure 6:**
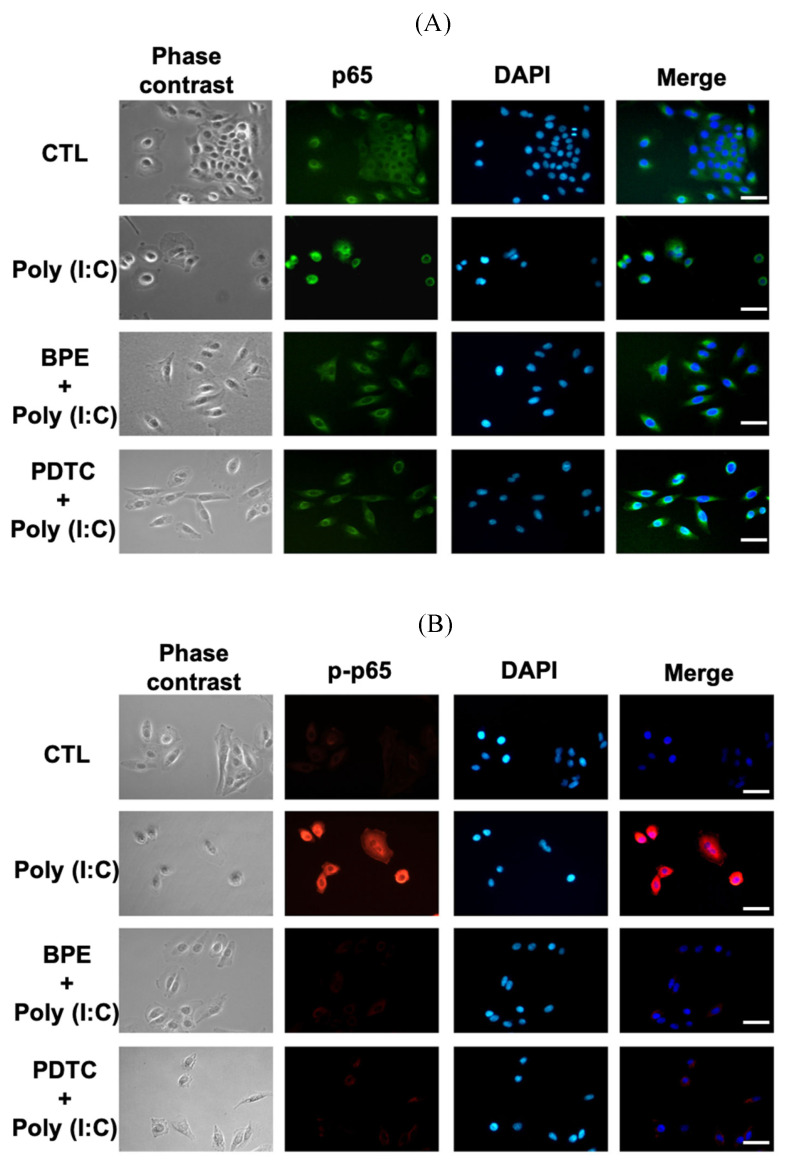
Representative immunofluorescence images showing effect of BPE on the nuclear translocation of NF-κB p65 in poly (I:C)-stimulated A549 cells. A549 cells were pretreated with BPE (1/6000 dilution), PDTC (10 μM) or PBS for 1 h and then incubated with 10 μg/mL of poly (I:C) for 4 h. The cellular localization of NF-κB p65 was then detected by antibody against (A) p65 and (B) phospho-p65 (p-p65), respectively, and visualized via fluorescent secondary antibody under fluorescence microscope. The nuclear was labeled with 4',6-diamidino-2-phenylindole (DAPI) (blue fluorescence). Scale bar is 50 μm.

Regarding the inhibitory effect of BPE on NF-κB activity shown in Figure 4B, we investigated whether BPE could also inhibit poly (I:C)-induced NF-κB activation in A549 cells. Immunocytofluorescence analysis for nuclear translocation of NF-κB p65 subunit was performed in poly (I:C)-primed A549 cells. As shown in Figure 6A, the green fluorescence-labeled p65 subunit distributed dominantly in the cytoplasm of control A549 cells. Priming with poly (I:C) induced nuclear translocation of p65 protein in A549 cells, as evidenced by the condensed green immunocytofluorescence intensity in nuclear regions (labeled by DAPI) (Figure 6A). As expected, pretreatment with BPE obviously inhibited this nuclear translocation of p65 protein as that done by PDTC (Figure 6A). This phenomenon was more clearly demonstrated by labeling the phosphorylated p65 protein (p-p65, active form of p65) with red fluorescence (Figure 6B). Only very slight red fluorescence could be seen in PBS-treated A549 cells, and priming with poly (I:C) dramatically increased the red fluorescence staining intensity, indicating intense activation of NF-κB signaling (Figure 6B). Pretreatment with BPE or PDTC clearly diminished the increased red fluorescence (Figure 6B), implying the drastic inhibition on NF-κB activity.

**Figure 7:**
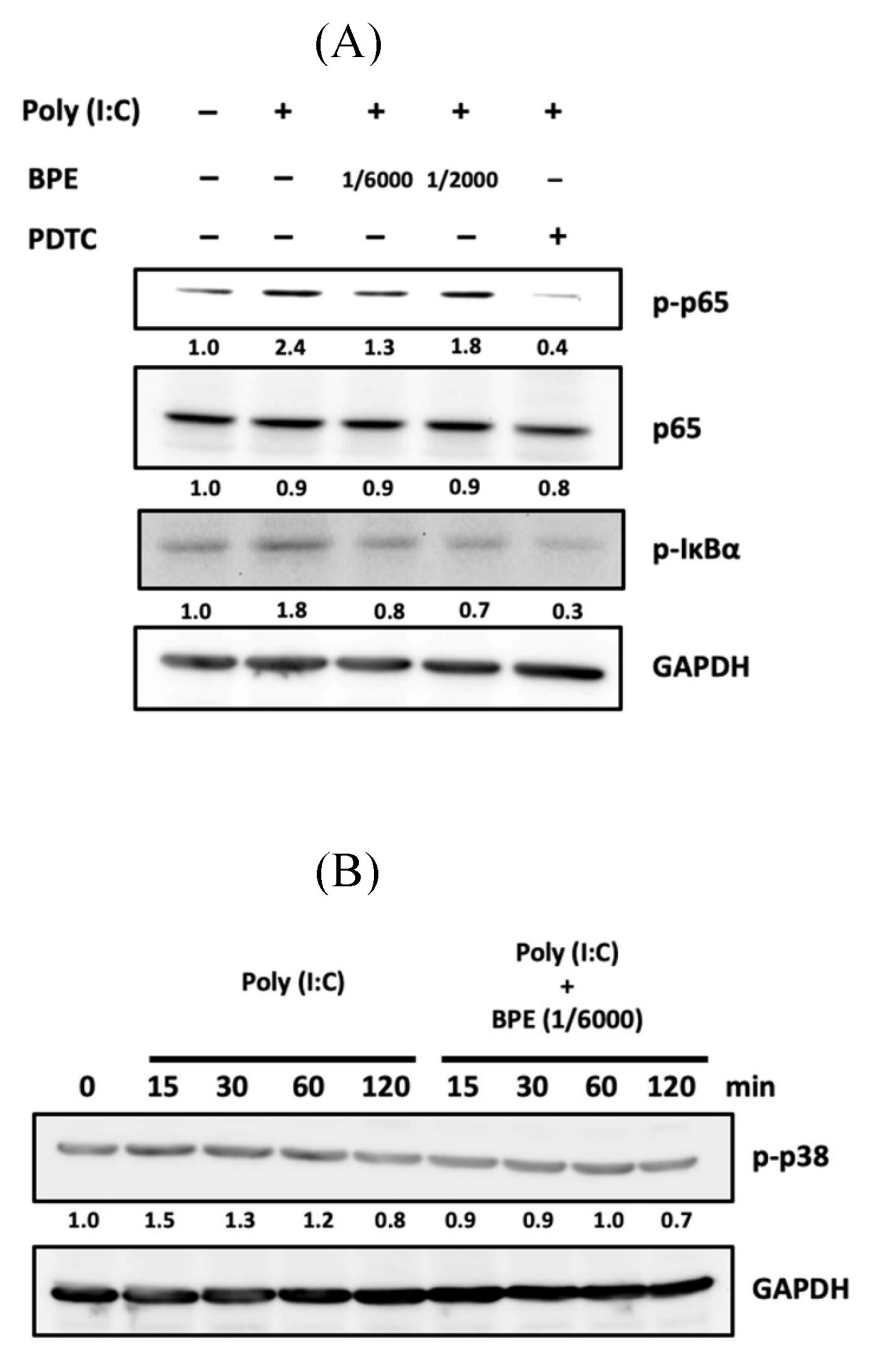
Effects of BPE on poly (I:C)-induced activation of NF-κB and p38 in A549 cells. (A) A549 cells were pretreated with BPE (1/6000 or 1/2000 dilution), PDTC (10 μM) or PBS for 1 h and then incubated with 10 μg/mL of poly (I:C) for 4 h. The protein level of NF-κB p65 (p65), phospho-p65 (p-p65), phospho-IκBα (p-IκBα), and GAPDH were detected by Western blots. After normalization to GAPDH, the fold changes of these protein levels were indicated below the bands. (B) A549 cells were pretreated with BPE (1/6000 dilution) or PBS for 1 h and then incubated with 10 μg/mL of poly (I:C) for 15, 30, 60, and 120 min, respectively. Western blot analysis of the phospho-p38 (p-p38) protein level was done. Numbers below the corresponding band indicate relative intensity after normalization to GAPDH.

Activation of NF-κB signaling requires phosphorylation and subsequent degradation of the inhibitory subunit of NF-κB alpha (IκBα) 32. Afterward, we examined the phosphorylation of IκBα by Western blot in poly (I:C)-stimulated A549 cells with or without BPE pretreatment. After primed with poly(I:C) for 4 h, an increased phosphorylated IκBα (p-IκBα) level was observed while activation of NF-κB was evidenced by the increased level of phosphorylated p65 subunit (p-p65) (Figure 7A). In agreement with the result of immunocytofluorescence shown in Figure 6, pretreatment with BPE suppressed the increase of both p-p65 and p-IκBα levels induced by poly(I:C) (Figure 7A). This result further confirmed the inhibitory effect of BPE on poly(I:C)-induced NF-κB activation in A549 cells.

It is reported that in pathogen (nontypeable Hemophilus influenza)-infected epithelial cells, p38 MAP kinase signaling mediates NF-κB activation 33. The phosphorylated (activated) p38 (p-p38) level in A549 cells was thus examined by Western blot at various time points (15, 30, 60, and 120 min) after priming with poly (I:C). As shown in Figure 7B, a transient increase of p-p38 occurred at 15 min after poly (I:C) stimulation, and pretreatment with BPE abrogated this transient increase of p-p38. In addition to suppressing NF-κB activation, this stabilization of p38 MAP kinase activity might also contribute to the inhibitory effect of BPE on poly (I:C)-induced inflammation.

## Discussion

From SARS-CoV, MERS-CoV to the recent COVID-19 (SARS-CoV-2), the fight against viral pneumonia remains continue and the treatment of patients with severe disease is still challenging and evolving. In the management COVID-19 patients, anti-inflammatory therapy plays a pivotal role in preventing further injury and organ damage or failure 10. According to the reported anti-inflammatory effects of *Musa* species products 15,17, we speculate the effects of a banana plant extract BPE on relieving viral inflammatory response in mice lung. By using the mouse model of pulmonary inflammation induced by RNA viral mimetic poly (I:C) 34, this study presents the anti-inflammatory effects of BPE in the aspects of modulating immune mediators production, suppressing immune cells infiltration, and inhibiting the prominent signaling molecule NF-κB.

BPE treatment substantially diminished poly (I:C)-induced pro-inflammatory cytokines and chemokines in BALF, implying its potential in attenuating the cytokine storm initiated by viral infection. Exaggerated overproduction of pro-inflammatory cytokines and chemokines (so called cytokine storm) has been observed in SARS-CoV, MERS-CoV, and the recent COVID-19-induced SARS-CoV-2 28. In hospitalized COVID-19 patients, cytokine storm was found to correlate with disease severity and unfavorable outcomes, which thus highlighted the implementation of anti-inflammatory treatment 28. It is believed that IL-6 play a key role to incite the inflammatory cytokine storm, which may cause eventual pulmonary fibrosis and organ failure 35. The significantly increased IL-6 level in COVID-19 patients was found to closely correlate with acute respiratory distress syndrome (ARDS) severity and outcome 28. Moreover, preliminary clinical data showed the effective treatment of severe and critical COVID-19 patients by a recombinant humanized anti-human IL-6 receptor monoclonal antibody tocilizumab, suggesting the potential therapeutic strategy via interfering of IL-6 35. Coincide with this IL-6 targeting strategy, the present study demonstrated substantial reducing effect of BPE on poly (I:C)-induced IL-6 production both *in vivo* (BALF and immunohistochemistry staining of lung section) and *in vitro* (A549 epithelial-like, WI-38 fibroblast, and RAW 264.7 macrophage-like cells). These IL-6 suppressing effects further suggest the possibility of BPE to reduce the aforementioned cytokine storm. In addition to the antibody against IL-6, a case series study reported that RANTES (chemokine ligand 5, CCL5)-specific antibody leronlimab clinically improved critically COVID-19 patients and reduced the elevated plasma IL-6 and RANTES 36. In line with this, BPE suppressed both elevations of RANTES and IL-6 in the BALF from poly (I:C)-treated mice, implying a compelling anti-inflammation effect it exerted.

*Musa* species products have been shown to suppress pro-inflammatory *IL-6* in isoproterenol-induced hypertrophy rat heart, lipopolysaccharide (LPS)-treated embryonic rat heart cell line 16, and the wounds of diabetic rats 15. Parallelly, other researchers reported that *Musa* species products inhibited pro-inflammatory NF-κB activity in rat oral mucosal wound 37 and in BALF cells from ovalbumin-stimulated mice 17. Yet, the effects of *Musa* species products on *IL-6* level and NF-κB activity were not simultaneously analyzed in those above studies. The present study demonstrated remarkable suppressing effects of BPE against viral mimetic poly(I:C)-induced NF-κB activation and IL-6 production in mice. The crucial role of NF-κB inhibition in reducing highly pro-inflammatory cytokine such as IL-6 has been recently highlighted for treatment of critical stage COVID-19 patients 38. Inhibition of NF-κB was found to suppress both virus- and LPS-induced cytokine storm, and was proposed as a potential strategy for critical COVID-19 patients' treatment 38. In contrast to blocking single targets of inflammatory cytokine cascade, inhibition of NF-κB pathway provides the potential to simultaneously inhibit multiple strongly pro-inflammatory cytokines and chemokines, as well as adhesion molecules increased during acute COVID-19 stages 38. In accordance with this notion, upon inhibiting NF-κB, BPE markedly suppressed the elevation of multiple pro-inflammatory cytokines and chemokines in the BALF from poly (I:C)-stimulated mice. Of note, impressively decreased mortality has been observed in COVID-19-infected patients treated by various approved medications with implicated NF-κB suppressing activity, such as Aspirin 39, Bruton tyrosine kinase inhibitors (e.g. Ibrutinib, Acalabrutinib) 40,41, Dexamethasone 42, and N-acetyl-cysteine 43. Nevertheless, treatments by above medications so far could not completely prevent the mortality of COVID-19 patients yet 43,44. The central role of NF-κB activation in the progression of acute pneumonia inflammation has been demonstrated by several studies 45. Considering the substantial effect of BPE on NF-κB inhibition, it might hopefully be used as a complimentary for overcoming the limitation of those aforementioned NF-κB therapeutics in the treatment of severe pneumonia inflammation.

A previous study by Panda et al. had showed the antiviral activity of different plant parts of banana (*Musa spp.*) 46. In addition to aforementioned anti-inflammatory effects, BPE might also exhibit antiviral activity upon treatment of viral pneumonia. Compared to other natural plant extracts with antiviral or anti-inflammatory activity alone, treatment with BPE might exhibit both activities to combat viral pneumonia. It is worthy to investigate the antiviral activities of BPE in the future work for further demonstrating its potential in integrative treatment or prevention of viral pneumonia.

Like that reported in a previous study 47, poly (I:C) elevated the mRNA levels of both* IL-6* and *IL-10* in RAW 264.7 cells cultured *in vitro.* However, in the mouse model of this study, poly (I:C) challenge elevated pro-inflammatory IL-6 but diminished the anti-inflammatory IL-10 protein levels in lung tissue and BALF evidenced by immunohistochemistry and ELISA analyses, respectively. The mechanism underlying this unexpected result is unclear. Intriguingly, treatment with BPE not only repressed the elevated IL-6 but also restored the diminished IL-10 in the lung tissues and BALFs form poly (I:C)-stimulated mice. Upregulating IL-10 production was reported to ameliorate bacterial outer membrane vesicle-induced sepsis in mice 48, thus raising the possibility that the increased IL-10 contributed to BPE-mediated anti-inflammation effects in this study. However, besides IL-10's classical anti-inflammatory actions, a nonclassical pro-inflammatory effects of IL-10 was also proposed to explain the drastic early increased IL-10 in severe cases of COVID-19 49. Further mechanistic studies will be needed to investigate the exact role of BPE-restored IL-10 in treating viral pneumonia inflammation.

On the other hand, repressing the activity of pro-inflammatory cytokines has ameliorated diabetic wound healing in both animal models and humans 50. Human diabetic foot ulcers have decreased IL-10 level 50,51, and increasing IL-10 was proposed as an interesting option to improve healing of diabetic wounds 50. A previous study by Cheng et al. demonstrated that wound healing in diabetic rats was promoted by this water-soluble extract of *Musa paradisiaca* (Linn), BPE 15. The restored IL-10 by BPE found in this study might also occur in Cheng et al.'s study and participate in promoting wound healing in diabetic rats. The IL-10 restoring effect of BPE might be useful for maintaining physiological balance of host inflammatory response. Nevertheless, like the aforementioned paradoxical role of IL-10 in viral pneumonia inflammation, a recent study showed that IL-10 level was increased in diabetic wound during the acute phase of healing, and blocking IL-10 signaling during this phase stimulated healing, accompanied by increased scarring 52. Given these conflict views of IL-10 in anti-inflammation 49 and wound healing 52, a more nuanced appreciation of BPE-restored IL-10 is needed for its application to adequately manipulate IL-10 in a contextualized manner.

## Conclusions

**Scheme 1:**
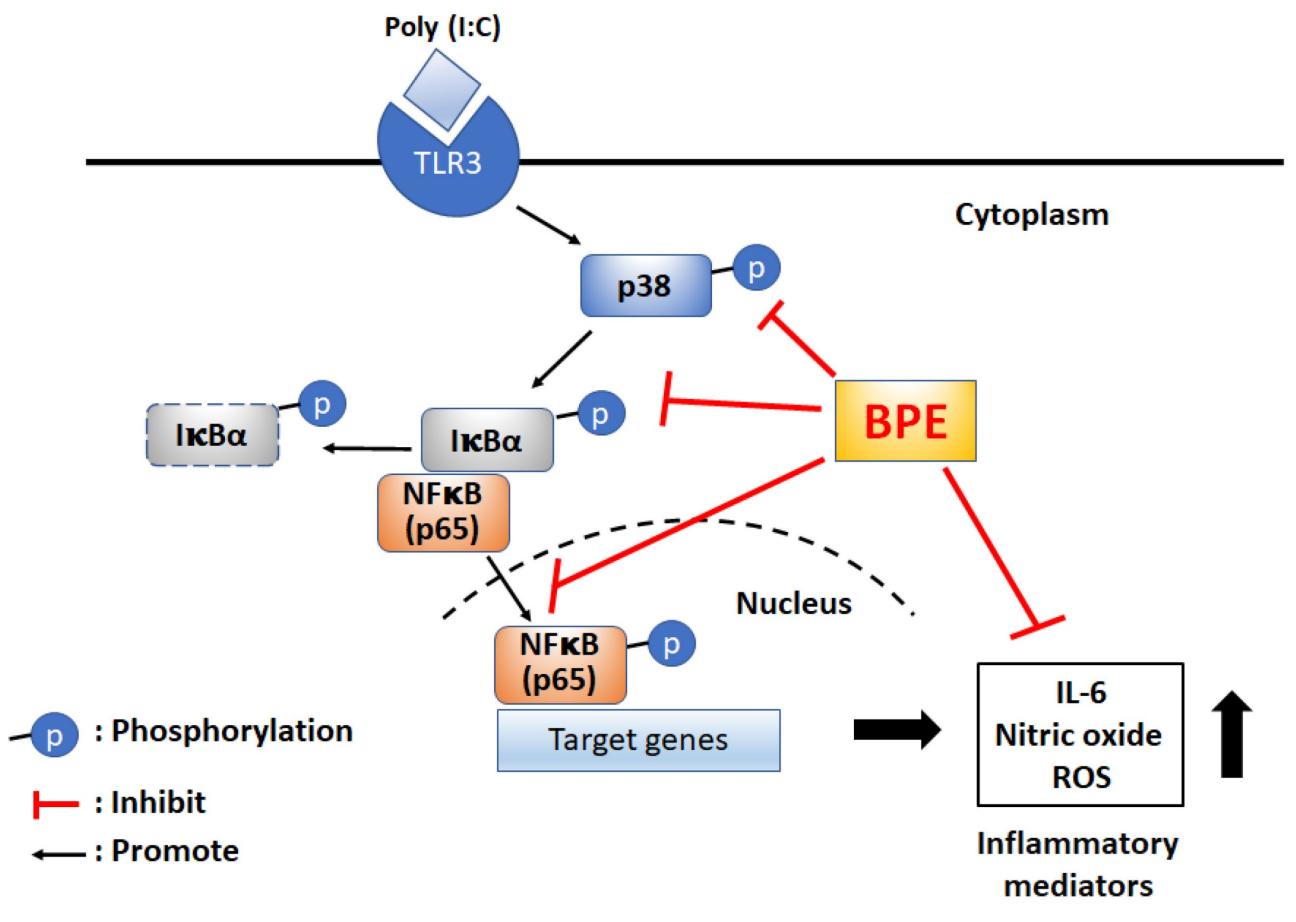
Proposed possible molecular mechanisms of BPE to suppress poly (I:C)-induced inflammatory responses. BPE: banana plant extract, TLR3: Toll like receptor 3, ROS: reactive oxygen species.

For the first time, the remedial effect of *Musa* species product, BPE, was demonstrated in viral mimetic poly(I:C)-induced acute pulmonary inflammation in mice. The possible underlying mechanisms were proposed as shown in scheme 1. Experimental results of this study unveil the capability of BPE to suppress poly(I:C)-induced activation of NF-κB and p38 MAPK signaling as well as the subsequent productions of IL-6 and pro-inflammatory mediators. Some effects of BPE observed in this study were not dose-dependent. This phenomenon was also shown in a previous study describing the wound healing promoting effect of BPE in diabetic rats 15. It might due to the complex components contented in BPE and the uncertain interactions between the components at various diluted concentrations. Further studies are under way to address this issue. As such, further satisfactory preclinical assessment to optimize the dosage, start, and duration of BPE treatment is required to evaluate its safety and efficacy as an integrative adjuvant for improving the management of viral pulmonary inflammation.

## Supplementary Material

Supplementary table.
