# Supplementary material for: Inhibition of Polyinosinic-Polycytidylic Acid-Induced Acute Pulmonary Inflammation and NF-κB Activation in Mice by a Banana Plant Extract
Source: Int J Med Sci. 2024 Jan 1;21(1):107–22. doi: 10.7150/ijms.88748 (PMC10750330; doi:10.7150/ijms.88748)
Supplement: Supplementary file 1 — Supplementary table. [file ijmsv21p0107s1.pdf]

Supplementary table

Supplementary Table 1 (Table S1). Phytochemical and mineral contents in BPE

No.	Test Item	Test Unit	Test Results
1	Flavone	mg/100mL	177.5
2	Polyphenol	mg/100mL	60.5
3	Polysaccharide	mg/100mL	433.2
4	Anthrone	mg/100mL	10.0
5	Hydroxyanthraquinone	mg/100mL	13.3
6	Triterpenoids	mg/100mL	22.5
7	Diterpenoids	mg/100mL	17.7
8	Steroids	mg/100mL	6.5
9	Potassium	mg/100mL	55.2
10	Sodium	mg/100mL	507.7
11	Lupine	mg/100mL	100.5
12	Selenium	mg/100mL	56.5
13	Anthraquinone	mg/100mL	76.4
14	Palmitic acid	mg/100mL	88.6
15	β-Sitosterol	mg/100mL	3.5
16	Amino acid	mg/100mL	355.8
17	Coumarin	mg/100mL	61.0
18	Saponin	mg/100mL	41.3
